# Etymologia:***Bordetella pertussis***

**DOI:** 10.3201/eid1608.ET1608

**Published:** 2010-08

**Keywords:** etymology, Etymologia, bordetella pertussis, Jules Bordet, aerobic coccobacilli, Octave Gengou

## [bor′′-də-tel′ə pər-tus′is]

Named for Belgian bacteriologist Jules Bordet, members of the genus *Bordetella* are small, gram-negative, aerobic coccobacilli that infect the respiratory epithelium in mammals. In 1906, Drs Bordet and Octave Gengou succeeded in isolating and cultivating the bacterium, later called *Bordetella pertussis* (from Latin *per*, intensive, and *tussis*, cough), which causes whooping cough, a deadly disease in young children. For this work and his pioneering immunologic studies, Dr Bordet was awarded the Nobel Prize in Physiology or Medicine in 1919.

**Source:** Bordet J, Gengou O. Le microbe de la coqueluche. Ann Inst Pasteur (Paris). 1906;20:731–41. http://nobelprize.org; Dorland’s illustrated medical dictionary, 31st edition. Philadelphia: Saunders Elsevier; 2007.

